# Risk of Treatment Failure and Death after Ablation in Hepatocellular Carcinoma Patients—A Multiparametric Prediction

**DOI:** 10.3390/cancers15133269

**Published:** 2023-06-21

**Authors:** Sergio Muñoz-Martínez, Victor Sapena, Ángeles García-Criado, Anna Darnell, Alejandro Forner, Ernest Belmonte, Marco Sanduzzi-Zamparelli, Jordi Rimola, Alexandre Soler, Neus Llarch, Gemma Iserte, Ezequiel Mauro, Carmen Ayuso, Jose Rios, Jordi Bruix, Ramon Vilana, María Reig

**Affiliations:** 1 Barcelona Clinic Liver Cancer (BCLC) Group, Institut d’Investigacions Biomèdiques August Pi i Sunyer (IDIBAPS), 08036 Barcelona, Spain; sergio.munoz@vhir.org (S.M.-M.);; 2 Centro de Investigación Biomédica en Red en Enfermedades Hepáticas y Digestivas (CIBEREHD), 28029 Madrid, Spain; 3 Faculty of Medicina, University of Barcelona, 08007 Barcelona, Spain; 4 Department of Clinical Pharmacology, Medical Statistics Core Facility, Hospital Clinic of Barcelona, 08036 Barcelona, Spain; 5 Biostatistics Unit, Faculty of Medicine, Autonomous University of Barcelona, 08193 Barcelona, Spain; 6 Liver Oncology Unit, Radiology Department, CDI, Hospital Clínic of Barcelona, 08036 Barcelona, Spain; 7 Liver Oncology Unit, Liver Unit, ICMDM, Hospital Clinic of Barcelona, 08036 Barcelona, Spain

**Keywords:** hepatocellular carcinoma, ablation, comorbidities, elderly, clinical decision-making, survival, pattern of progression

## Abstract

**Simple Summary:**

Patients with early-stage BCLC 0/A hepatocellular carcinoma (HCC) who are not candidates for liver transplantation or resection are treated with percutaneous ablation according to guidelines. Nevertheless, these patients are at high risk of HCC recurrence and physicians must apply different criteria to choose the salvage treatment as part of the Clinical Decision-Making process. This study analyzed the outcome of 225 BCLC 0/A HCC patients treated with ablation, focusing beyond the classical factors of tumor burden, liver function, and/or performance status. We found that the risk of death is two times higher (HR 2.0) if the comorbidities prevent further sequential locoregional or systemic treatments. The data in this study provide significant and useful prognosis information for physicians and provide valuable information to researchers involved in clinical practice and research by adding granularity to the evolutionary events of HCC patients with recurrence after percutaneous ablation.

**Abstract:**

Background: Ablation is a first-line treatment for Barcelona Clinic Liver Cancer (BCLC)-0/A hepatocellular carcinoma (HCC). However, there are scarce data about patients’ outcomes after recurrence. The present study evaluates the impact of patient and tumor characteristics at baseline and at recurrence on the Clinical Decision-Making process. Methods: We evaluated BCLC-0/A patients treated with percutaneous ablation from January 2010 to November 2018. Clinical and radiological data such as age, tumor location at ablation, pattern of recurrence/progression, and comorbidities during follow-up were registered. Tumor location was divided into ‘suboptimal’ vs. ‘optimal’ locations for ablation. The Clinical Decision-Making was based on tumor burden, liver dysfunction, or comorbidities. The statistical analysis included the time-to-recurrence/progression, censoring at time of death, date of last follow-up or liver transplantation, and time-to-event was estimated by the Kaplan–Meier method and Cox regression models to evaluate the risk of an event of death and change of treatment strategy. Results: A total of 225 patients [39.1% BCLC-0 and 60.9% BCLC-A] were included, 190 had unifocal HCC and 82.6% were ≤3 cm. The complete response rate and median overall survival were 96% and 60.7 months. The HCC nodules number (Hazard Ratio—HR 3.1), Child-Pugh (HR 2.4), and Albumin-Bilirubin score (HR 3.2) were associated with increased risk of death during follow-up. HCC in ‘suboptimal location’ presented a shorter time to recurrence. When comorbidities prevented further loco-regional or systemic treatment, the risk of death was significantly increased (HR 2.0, *p* = 0.0369) in comparison to those who received treatment. Conclusions: These results expose the impact of non-liver comorbidities when considering treatment for recurrence after ablation in the real-world setting and in research trials. Ultimately, we identified an orphan population for which effective interventions are needed.

## 1. Introduction

The landscape of hepatocellular carcinoma (HCC) has changed in the setting of systemic treatment, but also in the interventional field. Super-selective intra-arterial therapies or external radiotherapy approaches are proposed as competitive options for ablation in early-stage HCC [1,2]. Nevertheless, there are no robust data on prioritizing those treatments, and ablation is still the first recommended option in most of the International Clinical Guidelines for Barcelona Clinic Liver Cancer (BCLC)-0 or A who are not candidates for liver transplantation (LT) or surgery [1,1,3,4,5]. Complete response (CR) is a predictor of improved overall survival (OS) [6,7]. At the time of recurrence, physicians apply a multiparametric approach where the characterization of additional parameters is routinely applied and leads to a potential indication of salvage treatment. However, there are no data about what parameters dictate the decision to treat or not, while there is scarce information about the proportion of patients left untreated because of whatever reason. There is one unicentric Netherlands cohort [8] and two Asian cohorts [9,10] that reported patients in whom the recurrence is unamenable to repeat ablation. However, there are limited data about evolutionary events after HCC ablation (and re-treatments) until reaching a change of treatment strategy. This information is relevant both for understanding what the real-world management of HCC patients is, and also to inform the design and outcome assumptions in research trials in the adjuvant setting. Recurrence-free survival is a commonly accepted primary end-point in such studies but if they suffer from an imbalance in the proportion of patients treated at recurrence, the mandatory overall survival data may be heavily flawed and contradict any finding about recurrence-free survival [11,12,13,14,15]. Recently, the IMBRAVE 50 trial testing the Atezolizumab–Bevacizumab combination to reduce the risk of death has been announced positive [16] and when data become public, there will be a major need to critically inspect the data.

The Barcelona Clinic Liver Cancer 2022 strategy incorporated a specific chapter that highlights the role of the patient’s profile, as well as the evolutionary events at the time of defining the first and subsequent treatments [1]. This Clinical Decision-Making process is the result of the multiparametric analysis of objective factors, some of which are beyond the BCLC stage itself, such as HCC location, patient age, or pattern of HCC recurrence/progression, and subjective factors such as patient values and treatment availability.

Studies on HCC ablation usually report the evolutionary events until death to evaluate overall survival. However, in this study, we report an HCC cohort treated with ablation as the first strategy, documenting all the evolutionary events and focusing on detailing all the reasons that define the re-treatments strategy and the reason for the failure in the ablation treatment strategy.

This study analyzed the outcome of HCC patients treated with ablation, focusing on factors beyond tumor burden, liver function, and/or performance status. The age (elderly vs. non-elderly) at HCC treatment, HCC location, pattern of recurrence/progression after ablation, and competing risk factors related to comorbidities were also analyzed. This study identifies an orphan population, which is the group of patients who do not receive HCC-specific treatment due to comorbidities and for which effective interventions are needed.

## 2. Patients and Methods

This is a retrospective cohort study that evaluated BCLC-0/A HCC patients treated with percutaneous ablation either by radiofrequency ablation (RFA) or microwave ablation (MWA) from January 2010 to November 2018. Inclusion criteria: (1) patients older than 18 years, (2) HCC diagnosed according to the American Association for the Study of Liver Diseases (AASLD) guidelines [17,18,19], and (3) patients that underwent percutaneous ablation (RFA or MWA) as first-line therapy for their HCC. Exclusion criteria: Patients with a diagnosis of cancer different from HCC or without the minimum information required for the analysis (See Data Collection section). The liver disease etiology was determined in every case by evaluating the clinical history (search for metabolic risk factors, risk factors for viral hepatitis, alcohol intake, family liver disease history), laboratory analysis for viral hepatitis (Hepatitis C virus, Hepatitis B virus), and autoimmune autoantibodies. In those patients with suspicion of metabolic diseases, we also add ferritin levels and ceruloplasmin serum levels. If there was no clear liver disease etiology, a liver biopsy was performed. Data collection was performed from clinical history and registered in a database created specifically for this study (See the registered variables in Supplementary Materials S1). The study was approved by the Institutional Review Board (HCB/2019/0737 and HCB/2022/0292).

### 2.1. Study Definitions

***Elderly:*** Elderly was defined by the conventional 65-year cut-off [20,21,22].

***Locations:*** HCC location was divided into two categories according to the evaluation of the easiness of access, potential risk of complications, or locations that had been reported in previous studies to have a higher local recurrence rate [23,24,25,26,27,28]:

*Suboptimal location:* At least one HCC nodule was in one of the following areas: anterior subcapsular, hepatic dome, or in contact with or at <1 cm proximity to the heart, gastrointestinal tract, gallbladder, large blood vessels, or hepatic hilum.

*Optimal location*: All other locations.

### 2.2. Ablation Techniques Used in the Study

Percutaneous ablation procedures (RFA and MWA) were performed percutaneously and guided by ultrasound using local anesthesia and conscious sedation. RFA was performed using one of 3 possible pieces of equipment: an RF 2000 system electric generator with an expansible thermal needle, RadioTherapeutics Corp., Mountain View CA; a Radionics, Inc., Burlington, MA electric generator with a 17 gauge (G) monopolar electrode and a 2 or 3 cm internally cooled-tip needle; and an AMICA electric generator, Hospital Service, Italy with a monopolar electrode with a 2–3 cm cooled-tip 17 G needle. The applied power could vary between 100 and 150 watts (W) with a median treating time of 15–20 min. The MWA procedure was also performed percutaneously and guided by ultrasound using local anesthesia and conscious sedation. The AMICA equipment (Hospital Service, Aprilia, Noale, Italy) was used with a 2450 MegaHertz (MHz) energy generator and a thermocoagulation electrode with an internally cooled 16 G needle. The applied power was between 60 and 80 W, with an average of 6 to 10 min duration. In both procedures, the needle tract was cauterized by withdrawing the electrode while hot to prevent hemorrhagic adverse events and potential HCC dissemination. The treatment is chosen based on the HCC characteristics, usually performed in one procedure session and during a 24-h hospitalization surveillance for potential adverse events related to the procedure.

Co-adjuvant percutaneous ethanol injection (PEI) was performed in selected patients considered to have a suboptimal location HCC that would benefit from PEI as an adjunct to the percutaneous ablation with RFA of MWA to achieve CR and to avoid the risk of contiguous structures damage. The decision to perform it depends on the radiologist’s evaluation, which considers the HCC characteristics, mainly the presence or not of a suboptimal HCC location. The PEI procedure was performed by inserting a 22 G needle directly into the HCC nodule and injecting 100% alcohol. The procedure could be repeated over different days according to the necrosis achieved.

Response assessment was evaluated by contrast-enhanced ultrasound (CEUS) at months 1 and 3, and magnetic resonance (MR) or computerized tomography (CT) at month 6 and thereafter every 6 months. CR was registered if there was an absence of tumoral activity in a dynamic contrast-enhanced radiological test or the image visualized necrosis includes all the tumor areas after the ablation. Failure to achieve CR was considered as no-CR [29,30].

### 2.3. Pattern of Recurrence/Progression after the First Percutaneous Ablation

Intrahepatic recurrence was divided into—*Local recurrence* (*LR*): Unequivocal hyperenhancing lesion in the ablated region that had previously achieved CR and—*New Intrahepatic lesion* (*NIH*): New nodule ≥ 1 cm in a different location to the previously treated nodule. Extrahepatic progression was registered upon detection of a new lesion outside the liver (new extrahepatic lesion: NEH) or new vascular and/or biliary tract invasion. Time to intrahepatic recurrence or extrahepatic progression was defined as the time between the date of CR and the date of LR, NIH, and/or NEH.

Change of treatment strategy was defined by one of the following reasons: HCC not amenable to further ablation due to a technical reason such as visibility, accessibility, or tumor burden exceeding the BCLC staging criteria for ablation. This would be classified as a failure of treatment strategy reflecting the failure of ablation to control the disease. If the strategy after failure consisted of Best Supportive Care (BSC), patients were divided according to the reason for it: (1) symptomatic HCC progression, (2) patient comorbidities, or (3) liver dysfunction.

### 2.4. Follow-Up Protocol

Patients had clinical, laboratory, and radiological evaluation prior to each ablation procedure, and a CEUS one month after the procedure. Follow-up was planned according to the evaluation at the first month after the procedure: CR or no-CR. If the patient achieved CR at the first month, a 3-month CEUS follow-up was performed. If the patient had CR at month 3, the next control was at month 6 by MR. If the patient maintained patient maintained CR at month 6, radiological follow-up was performed every 6 months for 2 years with MR. After the 2-year follow-up with CR, the patient reverted to a 6-month ultrasound follow-up. Clinical and laboratory evaluation was performed at the time of radiological control. If the patient had no-CR in the ablated lesion at the 1st month of follow-up control, the patient was retreated with ablation if baseline criteria for ablation were maintained. If the patient had new lesions in subsequent follow-up controls plus preserved liver function, performance status, and tumor burden criteria for ablation, re-treatment with ablation was evaluated. Adverse events and/or complications were evaluated according to severity, graded according to Clavien-Dindo classification [31] and according to the Society of Interventional Radiology definition [32,33].

### 2.5. Statistical Analysis

Continuous variables were expressed as median and interquartile range [IQR: 25th–75th percentiles] and absolute ranges (minimum, maximum). Categorical or ordinal data were expressed as absolute frequency and percentages (%). Group comparisons were performed using a Mann–Whitney U test for continuous or ordinal variables, and Fisher’s exact test for categorical variables. Balance assessment between optimal location HCC vs. suboptimal location HCC patients was performed by means of standardized differences (STD). STD > 10% indicates unbalance [34]. Time-to-recurrence/progression was defined as the time between the date when the patient achieved CR and the date of recurrence/progression while censoring at the time of death and the date of the last follow-up or transplant. Since ablation in patients ultimately transplanted was used as bridging, such an instance was not considered a change of strategy. Time-to-event for each outcome was expressed as median and 95% confidence interval (95% CI), estimated by the Kaplan–Meier method. Survival curves were compared using the log-rank test. Cox regression models were performed, estimating Hazard Ratios (HR) and their 95% CI, to evaluate the risk of event for death and change of treatment strategy. Post-baseline parameters were considered as time-dependent factors. The level of significance was set at 5% (two-sided). All statistical analyses were performed using SAS 9.4 software (SAS Institute, Cary, NC, USA).

## 3. Results

### 3.1. Baseline Characteristics and Percutaneous Ablation Procedure

Between January 2010 and November 2018, 384 HCC patients were treated with ablation at our Institution; 225 met the inclusion criteria and were included in this analysis. The excluded patient’s summary is detailed in Supplementary Materials S2. Table 1 summarizes the baseline characteristics of the cohort study: 88 (39.1%) patients were BCLC-0 and 137 (60.9%) were BCLC-A. Median alpha-fetoprotein (AFP) was 7 ng/mL [IQR 4–19] (range 1–2928) in the whole cohort. Ninety patients (40%) had an AFP beyond the normal range (≥10 ng/mL) and their median AFP was 22 ng/mL [IQR 15–41] (min–max 15–2928).

Table 2 shows the baseline characteristics according to HCC location (optimal vs. suboptimal location).

Supplementary Table S1 describes the distribution of albumin-bilirubin (ALBI) score according to Child-Pugh score. Supplementary Table S2 shows the distribution of patients according to tumor location. The standardized mean difference was >10% between these two groups as per etiology, patients treated with DAA, Child-Pugh, ALBI score, BCLC stage, AFP level (<10 ng/mLvs. ≥10 ng/mL), number of nodules, number of insertions, and combined ablative treatment.

**Figure 1:**
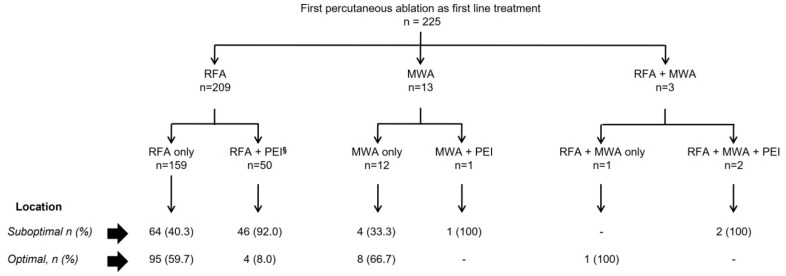
Distribution of the first percutaneous ablation procedure. §: 13 patients received RFA in the main nodule and PEI in a 2nd or 3rd nodule. The rest of the patients were treated with a combination of RFA and PEI in the same nodule. Abbreviations: HCC: Hepatocellular carcinoma; Hepato-CC: Hepato-Cholangiocarcinoma; RFA: Radiofrequency ablation; MWA: Microwave ablation; PEI: Percutaneous ethanol injection.

Figure 1 describes the distribution of the first ablation procedure by the lesion’s location. In brief, 209 (92.9%) patients were treated with RFA, 13 (5.8%) with MWA, and 3 (1.3%) with RFA plus MWA in the same session. One hundred and seventeen (52%) patients had a suboptimal location, and 49 (41.9%) of them had percutaneous ethanol injection (PEI) as an additional treatment in the first procedure (Supplementary Table S3).

**Outcomes:** After a median follow-up of 33.1 months [IQR 17.1–62.6], 106 (47.1%) patients had died and 57 (25.3%) had been transplanted. The median OS of the whole cohort was 60.7 months (95%CI 52.0–73.2).

Radiological outcome: Two-hundred sixteen out of the 225 patients achieved CR and 9 (4%) did not achieve CR at any time point (no-CR) (Supplementary Tables S4 and S5). Sixty-nine (31.9%) out of the 216 patients who achieved CR maintained CR until database lock and 147 (68.1%) developed recurrence/progression. Their median time to recurrence (TTR) was 18.1 months (95% CI 13.6–22.4). The patterns of recurrence registered were NIH lesions in 82 (55.8%) patients and local recurrence in 56 (38.1%). Additionally, nine (6.1%) patients developed NEH.

The median time to local recurrence was 6.8 months [IQR 4.1–14.6] and 12.2 months [IQR 6.1–24.4] for NIH lesions. The median time to extrahepatic progression was 9.7 months [IQR 3.5–28.3].

### 3.2. Failure of Ablation Strategy

**Figure 2:**
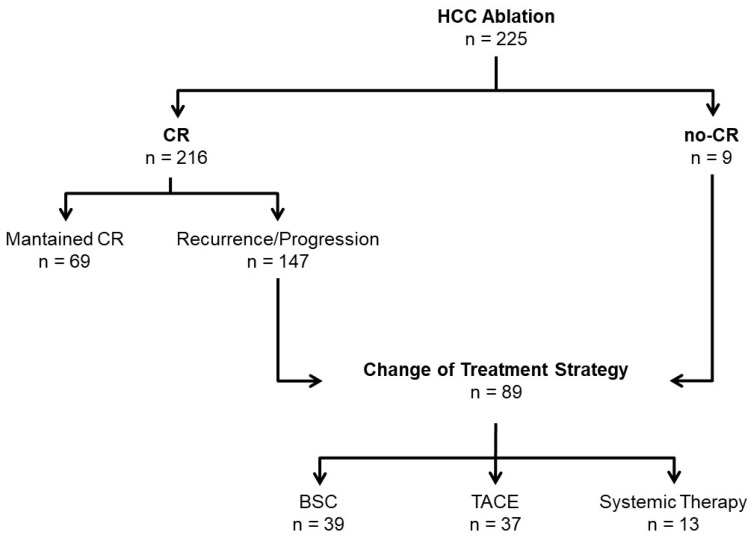
Flow-chart of treatment distribution until CR, no-CR, and change of treatment strategy. Abbreviations: CR: Complete response; no-CR: No complete response; BSC: Best Supportive Care; TACE: Transarterial chemoembolization.

Eighty-nine (60.5%) of the 147 patients who recurred presented failure of treatment strategy, meaning failure of ablation strategy to control the disease and leading to a change in treatment strategy. Forty-six of these patients (51.7%) received only one ablative treatment, 21 (23.6%) two, 15 (16.9%) three, and 7 patients more than three sessions before meeting the criteria of failure of treatment strategy (Figure 2). The median time to failure of ablation strategy was 22.6 months [IQR 11.1–45.3]. Sixty-six (74.2%) patients developed failure of ablation strategy within the first 3 years (Supplementary Table S6). Thirty-nine patients (43.8%) changed to BSC, 37 (41.6%) to transarterial chemoembolization (TACE), and 13 (14.6%) to systemic treatment.

The median time from first ablation to failure of ablation strategy in patients who were sequentially treated with BSC was 19.8 months [IQR 9.1–39.5]. The causes for selecting BSC were: 15 (38.5%) patients presented severe liver dysfunction, 15 (38.5%) suffered relevant comorbidities, and 9 (23.0%) presented symptomatic progression. Supplementary Table S7 shows the profile of patients who were treated with BSC after failure of ablation strategy due to relevant comorbidities. Among patients who received BSC, 13 were younger than 65 years but LT was not considering due to comorbidities (n = 5), extrahepatic progression (n = 4), intrahepatic recurrence/progression beyond Milan criteria (n = 2), absence of social support (n = 1), and drop-out after enlistment due to porto-pulmonary hypertension progression (n = 1).

*Impact of location:* Seventy-six (51.7%) patients with an HCC in the suboptimal location and 71 (48.3%) in the optimal location developed HCC recurrence. Local recurrence was observed in 31 (40.8%) with a suboptimal location and 25 (35.2%) with optimal location HCC; *p* = 0.5. The time to recurrence comparing patients with suboptimal vs. optimal HCC location was of 13.1 months [IQR 9.0–21.0] and 20.7 months [IQR 15.5–31.2], respectively; *p* = 0.0771. A shorter time to recurrence (13.1 vs. 20.7 months, *p* = 0.0771) and median time to change of treatment strategy (17.6 vs. 25.1 months; *p* = 0.0961) were observed in patients with HCC at suboptimal locations.

Among the 89 patients who changed treatment strategy due to meeting the criteria of failure of ablation strategy, 47 (52.8%) had the HCC in a suboptimal location and 42 (47.2%) were in an optimal one. Table 3 describes the sequential treatments for each subgroup. Median time to change of treatment strategy in the suboptimal location vs. optimal HCC location was 17.6 [IQR 8.3–29.7] months and 25.1 [IQR 12.5–43.4] months, *p* = 0.0961. The HR for ablation failure was 1.36 (95%CI, 0.9–2.1, *p* = 0.1462) in HCC suboptimal location (Yes vs. No) and 2.34 (95%CI, 1.3–4.3, *p* = 0.0066) in patients with more than one nodule (>1 vs. 1 nodule) (Table 4).

*Impact of age*: One-hundred and twenty (53.3%) of the patients were older than 65 years and 29 of them were treated with BSC after ablation due to liver dysfunction (n = 14; 48.3%), comorbidities (n = 10; 34.5%), or symptomatic HCC progression (n = 5; 17.2%). Table 5 shows the causes for recommending BSC in elderly vs. non-elderly patients: liver dysfunction [(6.7% vs. 93.3%), comorbidities (33.3% vs. 66.7%), and symptomatic progression (44.4% vs. 55.6%); *p* = 0.0608]. The rate of patients who received BSC after failure of ablation strategy was 29 (74.4%) and 10 (25.6%) in patients ≥65 years and <65 years, respectively.

Survival: The median OS of the whole cohort was of 60.7 months (95% CI 52.0–73.2). The 1-, 3- and 5-years survival rates were 94.8%, 72.8%, and 50.4%, respectively. Table 4 shows the risk factors of death in the whole cohort. The sole risk factors were number of lesions, Child-Pugh class, and ALBI score. The median OS of patients with HCC in a suboptimal location was 60.7 months (95%CI, 45.6–75.5) vs. 61.0 months (95%CI, 51.8–76.7) in the optimal HCC location, *p* = 0.209. Supplementary Materials S3 shows a sub-analysis performed excluding MW patients (n = 13).

Table 6 displays the risk factors for death in patients who received BSC after ablation. The HR was 2 (95%CI 1.04–3.82; *p* = 0.0369) for BSC due to comorbidities vs. TACE or systemic treatment; HR: 3.23 (95%CI 1.36–7.65; *p* = 0.0079) for BSC due to symptomatic progression vs. BSC for liver dysfunction; HR: 3.65 (95%CI 1.53–8.26; *p* = 0.0035) for BSC due to symptomatic progression vs. BSC for comorbidities and HR: 1.13 (95%CI 0.53–2.42; *p* = 0.7470) for BSC for liver dysfunction vs. BSC for comorbidities.

### 3.3. Adverse Events

After 371 ablation sessions, the complication rates by severity, graded according to Clavien-Dindo classification [31], were 26 (7.0%) grade I–II complications and 5 (1.3%) ≥ grade III. According to The Society of Interventional Radiology [32,33], 21 (5.7%) were mild, 3 (0.8%) moderate, 5 (1.3%) severe, 1 (0.3%) life-threatening, and 1 (0.3%) death. (See details in Supplementary Table S8). There were five grade III complications among the 225 patients. This represents a per-patient rate of 2.2% and corresponds to a 1.3% rate considering a total of 371 sessions. Complications included: one hemoperitoneum that was solved with selective arterial embolization; one hemothorax solved by arterial embolization; two liver abscesses that resolved with abscess drainage and antibiotics, one of them associated with intestinal perforation and the other with ischemic hepatitis; and one patient with severe liver decompensation that evolved to death. Four of these five patients had an HCC in a suboptimal location at the time of the ablation. There was only one case of seeding (0.3% considering the total of ablation sessions, which represents 0.4% of the total cohort) (See Supplementary Materials S4 for details on grade III adverse events and seeding cases).

## 4. Discussion

This study validates the response rate (96%), the competitive median OS results (60.9 months), and the 1-, 3-, and 5-year survival rates reported by ourselves and other authors in BCLC 0/A patients treated with percutaneous ablation [11,12,28,35,36,37,38,39,40,41,42]. Additionally, it adds key information about the value of factors that physicians usually consider in the Clinical Decision-Making process but for which there was no objective information to share with patients until this manuscript. Thereby, this is the first study that assessed the risk of death according to the comorbidities, liver function, or symptomatic progression at the time of developing HCC recurrence after receiving percutaneous ablation.

Our study characterizes the risk of death after ablation failure and survival while censoring at the time of liver transplantation. This involves the expected rate of intrahepatic recurrence or extrahepatic progression (68.1%), the rate of change of treatment strategy (60.5%) due to HCC recurrence not amenable to repeated ablation, and the rate of patients who developed HCC recurrence and received BSC (26.5%). Finally, it informs about the expected median time from achieving CR with ablation to change of treatment strategy (22 months). This item is relevant as a novel endpoint in studies where data about long-term survival would take years and is expected to become a surrogate for it [43]. The change of treatment strategy is the reflection of losing the clinical benefit of the treatment applied and opens the window of opportunity to propose unexplored sequential treatment schemes. Our data show that the reason for changing the treatment strategy is highly heterogeneous. This is relevant when assessing long-term OS in cohort studies and research trials with new agents, such as immune-oncology drugs aiming to prevent recurrence and, ultimately, cancer-related death. Not all recurrences are dismal and potential treatment upon recurrence significantly affects survival. These considerations will be key to understanding the results of the IMBRAVE050 trial [42] comparing the impact of the combination of atezolizumab and bevacizumab versus observation in HCC recurrence after successful treatment that has recently been announced as positive. According to that Clinical Trial, only 42 and 43 patients out of 668 included those receiving atezolizumab plus bevacizumab (AB) or active surveillance (AS) after ablation, respectively [42]. In addition, the rate of patients with single lesions was 70% and 73.8% in AB and AS, respectively. In our cohort, we had more single lesions, in 84.4% of patients, and the rate of patients who developed extrahepatic progression was 6.1% (median follow-up 33.1 months) and the median time to extrahepatic progression was 9.7 months [IQR 3.5–28.3]. Yun et al. also evaluated the rate of patients who developed extrahepatic progression after ablation and despite the fact that their cohort included 94% of patients with single lesions treated with ablation, and the rate of extrahepatic progression was 11.8% [44]. Thus, these data reflect the complexity in the analysis of the co-factors that affect the post-progression survival after ablation and the need for exploring deeply the results of the adjuvant scheme such as the AB, which was proposed in the IMBRAVE050 Clinical Trial. The results of the present study could be used as a reference when analyzing the outcome of patients who could be considered candidates for adjuvant treatment after ablation.

In our study, the number of nodules, as well as Child-Pugh and ALBI scores were associated with worse OS. As expected, the indication of BSC after ablation failure due to comorbidities was associated with an increased risk of death. After stratifying by cause of selecting BSC, the risk of death was significantly higher in patients who received BSC due to symptomatic progression than to liver dysfunction or comorbidities. These data offer the needed objective background at the time of sharing the treatment options and prognosis with patients or relatives. If treatment is not to be advised because of any reason, the expected prognosis can be stratified according to the cause of the decision.

HCC location and the patient’s age are the main discussion points when deciding on HCC treatment by multidisciplinary boards. However, data about their impact on patient outcomes after ablation are scarce regarding location [12,23,24,25,27,28]. In this cohort, neither the HCC location nor the age (elderly vs. non-elderly) at first ablation were identified as risk factors of OS, or as risk factors for the need to change treatment strategy. Interestingly, the rate of local recurrence was similar between HCC at a suboptimal location vs. an optimal one.

The fact that a large number of patients (43.8%) initially treated by ablation received BSC upon detection of tumor recurrence emphasizes the significant role of competing risk factors for death and the need to integrate them into the Clinical Decision-Making process. This multiparametric information is essential not only to offer the precise prognosis to patients but also to estimate the sample size and the number of patients that will be lost during the follow-up for causes different to HCC progression in the setting of Clinical Trials design.

The rate of patients who changed to BSC after ablation was significantly different according to age (25.6% in <65 years and 74.4% in ≥65 years). The causes for selecting BSC were also different in patients <65 years vs. ≥65 years [liver dysfunction patients (6.7% vs. 93.3%), comorbidities (33.3% vs. 66.7%), and symptomatic progression (44.4% vs. 55.6%); *p* = 0.0608]. Only one study in the ablation field has described the evolutionary events after first ablation [8]. That cohort included younger as well as more advanced patients than those that were included here. The incorporation of alternative HCC treatment options such as stereotactic body radiation therapy (SBRT) or systemic treatments without antiangiogenic drugs such as anti-PD1/PDL1 [45,46,47,48,49] that were not available during the study period may currently benefit elderly patients with preserved liver function and preserved performance status. The main limitation of the study is that it was a single institution retrospective study. To minimize the bias related to the nature of the study, data were reviewed systematically from the original medical reports of patients following a preset protocol for ablation in the BCLC group, and any uncertainties in the radiology reports were doubled checked with the Radiology Department.

## 5. Conclusions

In conclusion, this study validates the response rate and OS benefits in BCLC-0/A patients treated with ablation and characterizes the outcome of HCC patients after ablation. The risk of death after ablation failure varies according to the multiparametric assessment of the patients and is higher in patients who receive the best supportive care due to comorbidities, liver function, or symptomatic progression. Thus, a relevant proportion of patients with clearly different post-progression survival after treatment failure may hinder the long-term survival benefit of interventions that reduce recurrence as recognized by improved recurrence-free survival.

## Figures and Tables

**Table 1:** Baseline study cohort characteristics.

Patient Profile	n = 225
Age at diagnosis (years), median [IQR]	66 [57–74.6]
Gender (male), n (%)	144 (64)
**Etiology, n (%)**	
HCV ^a^	154 (68.4)
Enol	42 (18.7)
Others ^b^	11 (4.9)
HBV ^c^	11 (4.9)
Metabolic syndrome	7 (3.1)
Arterial hypertension, n (%)	104 (46.2)
Diabetes, n (%)	60 (26.7)
Patients treated with DAA, n (%)	55 (35.7) ^d^
Sustained Viral Response, n (%)	53 (96.4)
**Child-Pugh, n (%)**	
A	180 (81.8)
B	39 (17.7)
C	1 (0.5)
Non-cirrhotic	5
**ALBI score, n (%)**	
ALBI 1	192 (85.3)
ALBI 2	31 (13.8)
ALBI 3	2 (0.9)
BCLC, n (%)	
0	88 (39.1)
A	137 (60.9)
Nodule diameter (mm), median [IQR] (min–max)	22 [17–28] (10–50)
Median number of nodules, [IQR] (min–max)	1 [1,1] (1–3) ^d^
**Number of nodules, n (%)**	
1	190 (84.4)
>1	35 (15.6)
Unifocal HCC, n (%) ^e^	
≤2 cm	88 (46.3)
2–≤3 cm	69 (36.3)
>3 cm	33 (17.4)
Total bilirubin (mg/dL), median [IQR]	1.1 [0.8–1.6]
Albumin (g/L), median [IQR]	38 [35–41]
AFP (ng/mL), median [IQR] (min–max)	7 [3,4,6–19] (1–2928)
AFP (ng/mL), n (%)	
<10	135 (60)
≥10	90 (40)
INR, median [IQR]	1.2 [1.1–1.3]
Platelet count (×10^9^/L), median [IQR]	107 [70–150]

Abbreviations: IQR: Interquartile range; HCV: Hepatitis C virus; HBV: Hepatitis B virus; DAA: Direct-acting antiviral; ALBI score: Albumin-Bilirubin score; BCLC: Barcelona Clinic Liver Cancer; AFP: Alpha-fetoprotein; HCC: Hepatocellular carcinoma; HDV: Hepatitis D virus; INR: International Normalized Ratio. **^a^** 13 patients with HCV and alcohol etiology combination, 1 patient with HCV and autoimmune hepatitis, and 1 patient with HCV and HBV coinfection. **^b^ Others:** enol with metabolic associated liver disease (4), cryptogenic (n = 4); primary biliary cholangitis (n = 2); alcohol with hemochromatosis (n = 1). **^c^** Three HBV patients co-infected with HDV, and 4 patients with HBV and alcohol etiology combination. **^d^** Two patients with 3 HCC nodules. **^e^** Percentages over 190 unifocal HCC cases.

**Table 2:** Baseline characteristics according to HCC location.

Patients Profile	All Cohort (n = 225)	Optimal Location HCC (n = 108)	Suboptimal Location HCC (n = 117)	STD (%)
Age at diagnosis (Years), median [IQR]	66 [57–74.6]	66 [56.6–76.1]	66.6 [57.2–74]	2.0
Gender (male), n (%)	144 (64)	70 (64.8)	74 (63.2)	3.3
Etiology, n (%)				
HCV	154 (68.4)	75 (69.4) ^a^	79 (67.5) ^b^	31.3
Enol	42 (18.7)	21 (19.4)	21 (18.0)	
Others	11 (4.9)	2 (1.9) ^c^	9 (7.7) ^d^	
HBV	11 (4.9)	7 (6.5) ^e^	4 (3.4) ^f^	
Metabolic syndrome	7 (3.1)	3 (2.8)	4 (3.4)	
Patients treated with DAA, n (%)	55 (35.7)	24 (32)	31 (39.2)	15.2
Child-Pugh, n (%)				
A	180 (81.8)	90 (84.9)	90 (78.9)	23.0
B	39 (17.7)	15 (14.2)	24 (21.1)	
C	1 (0.5)	1 (0.9)	0 (0)	
Non-cirrhotic	5	2	3	
ALBI score, n (%)				
ALBI 1	192 (85.3)	97 (89.8)	95 (81.2)	38.0
ALBI 2	31 (13.8)	9 (8.3)	22 (18.8)	
ALBI 3	2 (0.9)	2 (1.9)	0 (0)	
Arterial hypertension, n (%)	104 (46.2)	52 (48.1)	52 (44.8)	6.7
Diabetes, n (%)	60 (26.7)	27 (25)	33 (28.4)	7.8
BCLC, n (%)				
0	88 (39.1)	47 (43.5)	41 (35)	17.4
A	137 (60.9)	61 (56.5)	76 (65)	
AFP (ng/mL), median [IQR] (min-max)	7 [3,4,6–19] (1–2928)	7 [4–14.5] (2–286)	7 [3,4,6–19] (1–2928)	7.8
AFP (ng/mL), n (%)				
<10	135 (60)	70 (64.8)	65 (55.6)	19.0
≥10	90 (40)	38 (35.2)	52 (44.4)	
Number of nodules, n (%)				
1 nodule	190 (84.4)	101 (93.5)	89 (76.1)	50.1
>1 nodule	35 (15.6)	7 (6.5)	28 (23.9)	
Type of treatment, n (%)				
RFA	209 (92.9)	99 (91.7)	110 (94)	42.6
MWA	13 (5.8)	8 (7.4)	5 (4.3)	
Combined RFA/MWA ^g^	3 (1.3)	1 (0.9)	2 (1.7)	
Number of insertions, median [IQR] (min–max)	0/1 [1] (1–3)	0/1 [1] (1–3)	0/1 [1] (1–3)	48.2
PEI performed as additional treatment, n (%)	53 (23.6)	4 (3.7) ^h^	49 (41.9)	100

Abbreviations: IQR: Interquartile range; STD: Standardized mean difference; HCV: Hepatitis C virus; HBV: Hepatitis B virus; DAA: Direct-acting antiviral; ALBI score: Albumin-Bilirubin score; BCLC: Barcelona Clinic Liver Cancer; AFP: Alpha-fetoprotein; RFA: Radiofrequency ablation; MW: Microwave ablation; PEI: Percutaneous ethanol injection; HDV: Hepatitis D virus; min: minimum; max: maximum. ^a^. 8 patients with HCV and enol combination and 1 patient with HCV and autoimmune hepatitis. ^b^. 5 patients with HCV and enol combination and 1 patient with HCV-HBV combination. ^c^. Enol with metabolic associated liver disease (2). ^d^. Cryptogenic (4); enol with metabolic associated liver disease (2), primary biliary cholangitis (2), enol with hemochromatosis (1). ^e^. 1 HBV patient co-infected with HDV. ^f^. 2 HBV patients co-infected with HDV. ^g^. Procedures not performed in the same nodule. ^h^. 3 patients with HCC nodule in posterior subcapsular location and 1 patient with HCC nodule in proximity to kidney.

**Table 3:** Sequential treatment options according to HCC location.

Change of Treatment Strategy (n = 89)	Optimal Location HCC (n = 42, 47.2%)	Suboptimal Location HCC (n = 47, 52.8%)
Best Support Care, n (%)	20 (47.6)	19 (40.4)
HCC treatment, n (%)	22 (52.4)	28 (59.6)
• TACE, n (%)	17 (77.3)	20 (71.4)
• Systemic treatment, n (%)	5 (22.7)	8 (28.6)

**Abbreviations**: HCC: Hepatocellular carcinoma; TACE: Transarterial chemoembolization.

**Table 4:** Univariate analysis of risk factors for Death and Change of Treatment Strategy.

Event	Parameter	Categories (Cat vs. Ref.)	Events/ Patients at Risk	Hazard Ratio (95%CI)	*p*-Value (Category)	*p*-Value (Parameter)
**All patients (n = 225)**						
**Death**	Age	≥65 vs. <65	67/119 vs. 39/106	0.97 (0.65–1.44)	0.8716	0.8716
	Age (cont.)	Increase of 1 year	106/225	1.01 (0.99–1.03)	0.3524	0.3524
	AFP (baseline)	Increase of 100 units	106/225	0.99 (0.86–1.12)	0.8273	0.8273
	BCLC	A vs. 0	67/137 vs. 39/88	1.43 (0.96–2.13)	0.0760	0.0760
	Child-Pugh	B or C vs. non-cirrhotic or A	22/40 vs. 84/185	2.39 (1.48–3.84)	0.0003	0.0003
	ALBI score	ALBI 2 vs. ALBI 1	18/31 vs. 87/192	3.22 (1.92–5.4)	<0.0001	<0.0001
		ALBI 3 vs. ALBI 1	1/2 vs. 87/192	2.43 (0.34–17.58)	0.3796	
	Number of nodules	>1 nodule vs. 1 nodule	17/35 vs. 89/190	3.1 (1.8–5.33)	<0.0001	<0.0001
	Suboptimal location	Yes vs. No	57/117 vs. 49/108	1.28 (0.87–1.87)	0.2111	0.2111
	Nodule diameter	>20–≤30 mm vs. 10–≤20 mm	48/92 vs. 43/99	1.4 (0.92–2.11)	0.1145	0.2791
		>30 mm vs. 10–≤20 mm	15/34 vs. 43/99	1.11 (0.62–2.01)	0.7265	
**Change of treatment strategy**	Age	≥ 65 vs. <65	55/119 vs. 34/106	0.98 (0.64–1.51)	0.9290	0.9290
	Age (cont.)	Increase of 1 year	89/225	1.00 (0.98–1.02)	0.7098	0.7098
	AFP	Increase of 100 units	89/225	1.10 (1.04–1.16)	0.0012	0.0012
	BCLC	A vs. 0	54/137 vs. 35/88	1.34 (0.87–2.06)	0.1823	0.1823
	Child-Pugh	B or C vs. non-cirrhotic or A	10/40 vs. 79/185	0.9 (0.46–1.74)	0.7440	0.7440
	ALBI score	ALBI 2 vs. ALBI 1	9/31 vs. 80/192	1.4 (0.7–2.83)	0.3433	0.6381
		ALBI 3 vs. ALBI 1	0/2 vs. 80/192	NE	-	
	Number of nodules	>1 nodule vs. 1 nodule	13/35 vs. 76/190	2.34 (1.27–4.32)	0.0066	0.0066
	Suboptimal location	Yes vs. No	47/117 vs. 42/108	1.36 (0.9–2.07)	0.1462	0.1462
	Nodule diameter	>20–≤30 mm vs. 10–≤20 mm	35/92 vs. 39/99	1.1 (0.7–1.74)	0.6824	0.3339
		>30 mm vs. 10–≤20 mm	15/34 vs. 39/99	1.57 (0.86–2.87)	0.1404	
**Patients with 1 nodule (n = 190)**						
**Death**	Age	≥65 vs. <65	59/106 vs. 30/84	1.08 (0.70–1.69)	0.7236	0.7236
	Age (cont.)	Increase of 1 year	89/190	1.01 (0.99–1.03)	0.2287	0.2287
	AFP	Increase of 100 units	89/190	0.99 (0.86–1.14)	0.8493	0.8493
	BCLC	A vs. 0	50/102 vs. 39/88	1.19 (0.78–1.82)	0.4142	0.4142
	Child-Pugh	B or C vs. non-cirrhotic or A	18/33 vs. 71/157	2.32 (1.37–3.91)	0.0016	0.0016
	ALBI score	ALBI 2 vs. ALBI 1	13/23 vs. 75/165	2.89 (1.6–5.25)	0.0005	0.0015
		ALBI 3 vs. ALBI 1	1/2 vs. 75/165	2.76 (0.38–20.1)	0.3150	
	Suboptimal location	Yes vs. No	44/89 vs. 45/101	1.13 (0.74–1.71)	0.5798	0.5798
	Nodule diameter	>20–≤30 mm vs. 10–≤20 mm	36/69 vs. 39/88	1.24 (0.78–1.96)	0.3599	0.6566
		>30 mm vs. 10–≤20 mm	14/33 vs. 39/88	1.09 (0.59–2.01)	0.7831	
**Change of treatment strategy**	Age	≥65 vs. <65	48/106 vs. 28/84	1.00 (0.62–1.60)	0.9910	0.9910
	Age (cont.)	Increase of 1 year	76/190	1.00 (0.98–1.02)	0.8141	0.8141
	AFP	Increase of 100 units	76/190	1.10 (1.04–1.16)	0.0013	0.0013
	BCLC	A vs. 0	41/102 vs. 35/88	1.17 (0.74–1.84)	0.5102	0.5103
	Child-Pugh	B or C vs. non-cirrhotic or A	8/33 vs. 68/157	0.85 (0.4–1.77)	0.6544	0.6544
	ALBI score	ALBI 2 vs. ALBI 1	6/23 vs. 70/165	1.22 (0.52–2.84)	0.6469	0.9003
		ALBI 3 vs. ALBI 1	0/2 vs. 70/165	NE	-	
	Suboptimal location	Yes vs. No	39/89 vs. 37/101	1.42 (0.9–2.23)	0.1297	0.1297
	Nodule diameter	>20–≤30 mm vs. 10–≤20 mm	26/69 vs. 35/88	1.00 (0.6–1.66)	0.9912	0.2148
		>30 mm vs. 10–≤20 mm	15/33 vs. 35/88	1.66 (0.9–3.07)	0.1032	
**Patients with 1 nodule and nodule ≤3 cm (n = 149)**						
**Death**	Age	≥65 vs. <65	45/86 vs. 26/63	0.98 (0.60–1.59)	0.9295	0.9295
	Age (cont.)	Increase of 1 year	71/149	1.01 (0.99–1.04)	0.2694	0.2694
	AFP	Increase of 100 units	71/149	1.62 (1.01–2.58)	0.0438	0.0438
	BCLC	A vs. 0	32/61 vs. 39/88	1.27 (0.79–2.04)	0.3164	0.3164
	Child-Pugh	B or C vs. non-cirrhotic or A	17/28 vs. 54/121	2.67 (1.53–4.63)	0.0005	0.0005
	ALBI score	ALBI 2 vs. ALBI 1	11/19 vs. 59/128	3.95 (2.03–7.7)	<0.0001	0.0002
		ALBI 3 vs. ALBI 1	1/2 vs. 59/128	2.9 (0.4–21.26)	0.2943	
	Suboptimal location	Yes vs. No	34/70 vs. 37/79	0.99 (0.62–1.58)	0.9617	0.9617
	Nodule diameter	>20–≤30 mm vs. 10–≤20 mm	32/61 vs. 39/88	1.27 (0.79–2.04)	0.3164	0.3164
**Change of treatment strategy**	Age	≥65 vs. <65	34/86 vs. 23/63	0.82 (0.48–1.40)	0.4641	0.4641
	Age (cont.)	Increase of 1 year	57/149	0.99 (0.97–1.02)	0.5468	0.5468
	AFP	Increase of 100 units	57/149	1.41 (0.77–2.58)	0.2666	0.2666
	BCLC	A vs. 0	22/61 vs. 35/88	0.97 (0.56–1.66)	0.8982	0.8982
	Child-Pugh	B or C vs. non-cirrhotic or A	7/28 vs. 50/121	1.01 (0.45–2.24)	0.9831	0.9831
	ALBI score	ALBI 2 vs. ALBI 1	4/19 vs. 53/128	1 (0.36–2.81)	0.9938	>0.9999
		ALBI 3 vs. ALBI 1	0/2 vs. 53/128	NE	-	
	Suboptimal location	Yes vs. No	28/70 vs. 29/79	1.15 (0.68–1.93)	0.6057	0.6057
	Nodule diameter	>20–<30 mm vs. 10–≤20 mm	22/61 vs. 35/88	0.97 (0.56–1.66)	0.8982	0.8982

**Abbreviations:** HCC: Hepatocellular carcinoma; BCLC: Barcelona Clinic Liver Cancer; 95%CI: 95% Confidence Interval; ALBI: Albumin-bilirubin score.

**Table 5:** Causes to recommend Best Supportive Care according to age.

Reason [n, (%)]	<65 Years (n = 10)	≥65 Years (n = 29)	*p*-Value (Dichotomized Parameter)	*p*-Value
Symptomatic progression	4 (44.4)	5 (55.6)	0.1970	0.0608
Liver dysfunction	1 (6.7)	14 (93.3)	0.0574	
Comorbidities	5 (33.3)	10 (66.7)	0.4633	

Abbreviations: HCC: Hepatocellular carcinoma; BSC: Best support care.

**Table 6:** Univariate analysis of risk factors for death according to the reason that defined the selection of Best Supportive Care as the post-ablation treatment.

Event	Parameter	Contrasts	HR (95%CI)	*p*-Value (Contrast)	*p*-Value (Parameter)
**Death**	Change of treatment strategy (td)	BSC for comorbidities vs. for HCC treatment	2 (1.04–3.82)	0.0369	<0.0001
		BSC for symptomatic progression vs. for liver dysfunction	3.23 (1.36–7.65)	0.0079	
		BSC for symptomatic progression vs. for comorbidities	3.65 (1.53–8.26)	0.0035	
		BSC for liver dysfunction vs. for comorbidities	1.13 (0.53–2.42)	0.7470	

Abbreviations: td: Time-dependent; HCC: Hepatocellular carcinoma; BSC: Best supportive care.

## Data Availability

The data that support the findings of this study are available on request from the corresponding author. The data are not publicly available due to privacy or ethical restrictions.

## Supplementary Materials

The following supporting information can be downloaded at: https://www.mdpi.com/article/10.3390/cancers15133269/s1, Supplementary Materials S1. Data collection of registered variables. Supplementary Materials S2. Description of the excluded patients. Supplementary Material S3. Sub-analysis of overall survival excluding patients that had MW as first percutaneous ablation (n = 13). Supplementary Materials S4. Location description of grade III adverse events and seeding events. Supplementary Table S1. ALBI distribution by Child-Pugh score. Supplementary Table S2: Location of the HCC classified as suboptimal location. Supplementary Table S3. Locations of the HCC classified as suboptimal location who received concomitant PEI and RFA. Supplementary Table S4. Tumor response at the first month after ablation. Supplementary Table S5. Baseline characteristics of patients who never achieved complete response. Supplementary Table S6. Rate of patients who changed the HCC treatment strategy at the 1st, 2nd, and 3rd year post-first ablation. Supplementary Table S7. Description of the comorbidities that prime BSC after presenting recurrence or progression post sequential ablation. Supplementary Table S8. Adverse events associated with ablation procedure by HCC location.
