# Supplementary material for: Risk of Treatment Failure and Death after Ablation in Hepatocellular Carcinoma Patients—A Multiparametric Prediction
Source: Cancers (Basel). 2023 Jun 21;15(13):3269. doi: 10.3390/cancers15133269 (PMC10339895; doi:10.3390/cancers15133269)
Supplement: Supplementary file 1 [file cancers-15-03269-s001.zip › cancers-2416741-supplementary.pdf]

Supplementary Material

Risk of treatment failure and death after ablation in hepatocellular carcinoma patients. A multiparametric prediction.

Sergio Muñoz-Martínez, Víctor Sapena, Ángeles García-Criado, Anna Darnell, Alejandro Forner, Ernest Belmonte, Marco Sanduzzi-Zamparelli, Jordi Rimola, Alexandre Soler, Neus Llarch, Gemma Iserte, Ezequiel Mauro, Carmen Ayuso, José Ríos, Jordi Bruix, Ramón Vilana, and María Reig

List of contents:

Page

Supplementary Material S1…………………………....………….2

Supplementary Material S2………………………….……….….…3

Supplementary Table S1. ……………………………………..….4

Supplementary Table S2. ……………………………………..….5

Supplementary Table S3. ……………………………………...…6

Supplementary Table S4. …………………………………..…….7

Supplementary Table S5. ……………..…………………………..8

Supplementary Table S6..…………………………………….….......9

Supplementary Table S7..……………………………………….........10

Supplementary Material S3…………………………………………11

Supplementary Table S8..……………………………………….........12

Supplementary Material S4..…………………..…………………...13

Supplementary Material S1. Data collection registered variables.

Baseline clinical characteristics including demographic variables, liver disease etiology, liver function by Child-Pugh classification and ALBI score, alpha-fetoprotein (AFP), performance status, and radiological pre-treatment HCC characteristics: localization, localization (suboptimal/optimal), and BCLC stage were registered.

Variables related to the ablation and evolutionary events: Radiological response, presence of intrahepatic recurrence or extrahepatic progression (Yes/No), pattern of recurrence/progression, retreatment in case of recurrence/progression (Yes/No), Change of Treatment Strategy based on the BCLC tumor board (Yes/No), cause of Change of Treatment Strategy, sequential treatments after Change of Treatment Strategy (transarterial chemoembolization [TACE], systemic treatment, Best Support Care [BSC]), cause of BSC (in cases where applied), adverse events related to the ablation procedure, death (Yes/No), and cause of death were registered.

Supplementary Material S2. Description of the excluded patients.

Between January 2010 and November 2018, 384 HCC patients were treated with ablation in our Institution, 225 met inclusion criteria and 159 patients were excluded. The exclusion criteria in these were: 88 for HCC treatment history; 35 for laparoscopic approach ablation; 15 for hepatocholangiocarcinoma diagnosis; 14 patients had an incomplete treatment (2 nodules treated at different times); and 7 had a diagnosis of cancer different to HCC (colon cancer in 2 patients, intrahepatic cholangiocarcinoma in 2 patients, hemangioendothelioma in 1 patient, lung cancer in 1 patient, and ovarian cancer in 1 patient).

Supplementary Table S1. ALBI distribution by Child-Pugh score.

Child-Pugh	ALBI Score n (%)			
	**1**	**2**	**3**	**Total**
**A**	172 (92.0)	8 (25.8)	0	180
**B**	15 (8.0)	23 (74.2)	1 (50)	39
**C**	0	0	1 (50)	1
**Total**	187	31	2	**220[¥]**

Abbreviations: ALBI: Albumin-bilirubin score. [¥]5 non-cirrhotic patients not shown.

Supplementary Table S2: Location of the HCC classified as suboptimal location.

Suboptimal location, n (%)	n = 117
Hepatic dome	45 (38.5)[a]
Proximity to large blood vessels	38 (32.5)[b]
Proximity to gastrointestinal tract	15 (12.8)[c]
Proximity to gallbladder	7 (6.0)
Anterior subcapsular	7 (6.0)
Hepatic hilum	4 (3.4)
Proximity to heart	1 (0.8)

HCC: Hepatocellular carcinoma.
Some patients had a 2nd nodule in a different suboptimal location:
[a.] 1 patient: proximity to gastrointestinal tract.
[b.] 4 patients: anterior subcapsular (2), hepatic dome (1), liver hilum (1).
[c.] 1 patient: anterior subcapsular.

Supplementary Table S3. Locations of the HCC classified as suboptimal location who received concomitant PEI and RFA.

Suboptimal location HCC	n = 49
Proximity to GI tract[a]	16
Hepatic dome	8
Proximity to large blood vessels[b]	9
Proximity to gallbladder	7
Anterior subcapsular[c]	5
Hepatic hilum	3
Proximity to heart	1

Abbreviations: HCC: Hepatocellular carcinoma; PEI: percutaneous ethanol injection.

[a.] 1 patient with a second nodule in the hepatic dome.

[b.] 1 patient with a second nodule anterior subcapsular and another patient with a second nodule in the liver hilum.

[c.] 1 patient with a second nodule in the proximity of the GI tract and another patient with a second nodule in proximity to blood vessels.

Supplementary Table S4. Tumor response at first month after ablation.

**Response [n, (%)]**	**n = 225**	**Unifocal HCC n = 190**	**Unifocal ≤3 cm HCC n = 157**
Complete response	196 (87.1)	168 (88.4)	146 (93.0)
No complete response	18 (8.0)[a]	14 (7.4)	5 (3.2)
Indeterminate	11 (4.9)[b]	8 (4.2)	6 (3.8)

Abbreviations: HCC: Hepatocellular carcinoma; CEUS: Contrast-enhanced ultrasound.

[a.] 12 patients achieved complete response after repeated ablation.

[b.] 8 patients were registered as complete response at the 3 months CEUS assessment after initial ablation.

Supplementary Table S5. Baseline characteristics of patients who never achieved complete response.

Patient	BCLC	Nodule max diameter (mm)	Number of nodules	Suboptimal location	Child-Pugh
1	0	15	1	No	B
2	A	16	2	Yes	A
3	A	23	1	Yes	A
4	A	32	1	Yes	A
5	A	34	1	No	A
6	A	40	1	Yes	A
7	A	46	1	No	A
8	A	29	1	No	B
9	A	40	2	Yes	B

Abbreviations: HCC: Hepatocellular carcinoma; BCLC: Barcelona Clinic Liver Cancer; mm: millimeters.

Supplementary Table S6. Rate of patient who change the HCC treatment strategy at 1st, 2nd, and 3rd year post-first ablation.

**Treatment**	**Year**		
	**1st (n=28),**	**2nd (n=23),**	**3rd (n=15),**
	**n(%)**		
**BSC**	13 (46.4)	9 (39.1)	6 (40.0)
Liver dysfunction	5	5	1
Comorbidities	4	2	4
Symptomatic progression	4	2	1
**TACE**	11 (39.3)	9 (39.1)	7 (46.7)
BCLC-B	4	4	4
BCLC-A and Treatment stage migration¥	7	3	3
No-CR after ablative re-treatment	-	2	-
**Systemic treatment**	4 (14.3)	5 (21.8)	2 (13.3)
BCLC-C	3	3	3
BCLC-B and Treatment stage migration	1	2	-

Abbreviations: HCC: Hepatocellular carcinoma; BSC: Best support care; TACE: Transarterial chemoembolization; No-CR: no complete response. ¥Due visibility or accessibility.

Supplementary Table S7. Description of the comorbidities which prime BSC after presenting recurrence or progression post sequential ablation.

Patient	Age when received BSC	Comorbidities
1	73	Ischemic stroke / Vasculopathy
2	77	Arterial hypertension / Advanced oral cancer (palliative CTx)
3	78	Arterial hypertension / Type 2 diabetes / Ischemic stroke / Biliary dilatation
4	81	AF / Arterial hypertension / Type 2 diabetes
5	82	Age / Leucopenia
6	83	Arterial hypertension / Decompensated heart failure / Intermittent claudication
7	84	Ischemic cardiopathy / Chronic kidney disease
8	85	AF / Arterial hypertension
9	85	Age
10	87	Arterial hypertension / Aortic Aneurism / COPD
11	55	Arterial hypertension / Type 2 diabetes / Chronic kidney disease / Uncontrolled psychiatric disorder
12	54	Severe obesity / COPD / OSAS (use of CPAP)
13	57	Severe obesity / Lung cancer
14	57	Severe aortic stenosis / TIPS / Type 2 diabetes / Chronic pancreatitis
15	60	Ischemic cardiopathy / Heart failure / AF / Type 2 diabetes

Abbreviations: CTx: Chemotherapy; AF: Atrial fibrillation; COPD: Chronic Obstructive Pulmonary Disease; OSAS: Obstructive Sleep Apnea Syndrome; CPAP: Continuous Positive Airway Pressure; TIPS: Transjugular Intrahepatic porto-systemic shunt.

Supplementary Material S3. Sub-analysis of overall survival excluding patients that had MW as first percutaneous ablation (n = 13).

- The overall survival of the whole cohort (n=212) was of 60.7 months (95%CI: 51.8 – 75.5).

- Eighty patients had RFA as first percutaneous ablation procedure and failure of treatment strategy, from which 63 patients died. The median OS in these patients was of 52.2 months (95%CI 39.5 - 65.4).

Supplementary Table S8. Adverse events associated to ablation procedure by HCC location.

AE*	Optimal location (n = 12)	Suboptimal location (n = 19)	Clavien-Dindo Classification	SIR AE Severity Scale
Pain	3[a]	7	I	Mild
Fever	1	1[b]	I	
Pain and Fever	0	1	I	
Nausea	0	1	I	
Skin electrical burn	1	0	I	
Syncope posterior to ablation	1	0	I	
Anesthesia related	2	0	I	
Perihepatic hematoma	1[a]	2[c]	I	
Colección intra-abdominal	1	1 [b]	II	Moderate
Bile duct injury	0	1	II	
Bacteremia and hepatic encephalopathy	1	0	II	Severe
Hemoperitoneum	0	2	II / III	
Liver abscess and intestinal perforation[d]	0	1	IIIa	
Hemothorax	1	0	IVa	
Ischemic hepatitis and liver abscess[e]	0	1	IVb	Life-threatening
Liver decompensation and death	0	1	V	Patient death

Abbreviations: SIR: The Society of Interventional Radiology; AE: Adverse Events.

* One patient diagnosed with seeding (not shown on table): Radiofrequency ablation with history of percutaneous biopsy of an optimal location HCC. The seeding was diagnosed 10 months after ablation, treated by laparoscopic resection, maintained complete response for 4 years and developed a new-intrahepatic recurrence.

[a.] One patient with pain and perihepatic hematoma (not in the same event).
[b.] One patient with fever and intraabdominal collection (same event).
[c.] One patient with perihepatic hematoma in 2 occasions (different events).
[d.] 27 mm HCC ablated.
[e.] 20 mm HCC ablated.

Supplementary Material S4. Location description of grade III adverse events and seeding event.

Five of these six patients had an HCC in a suboptimal location at the time of the ablation. One hemoperitoneum occurred in a binodular HCC close to the gastrointestinal tract and in anterior subcapsular location. The second hemoperitoneum involved an HCC with a near-vessel location. The patient with liver abscess and ischemic hepatitis had a XX cm HCC located in the hepatic dome. The patient with intestinal perforation and liver abscess had an HCC close to the gastrointestinal tract. The patient presenting severe liver decompensation leading to death had a HCC with a hepatic dome location. There was only one case of seeding (0.3% considering the total of ablations). This patient had a BCLC-0 HCC diagnosed by percutaneous biopsy in an optimal location. It was ablated by RFA and seeding was diagnosed 10 months after ablation. It was treated by laparoscopic resection. The patient-maintained CR for 4 years and eventually developed NIH recurrence.
